# Loneliness, online learning and student outcomes in college students living with disabilities: results from the National College Health Assessment Spring 2022

**DOI:** 10.3389/fpsyg.2024.1408837

**Published:** 2024-10-17

**Authors:** William Bevens, Sarah E. Stoeckl, Stephen M. Schueller, Jeongmi Kim, Biblia S. Cha, Cindy Chwa, Nicole A. Stadnick, Nakia C. Best, Dara H. Sorkin

**Affiliations:** ^1^Department of General Internal Medicine, University of California, Irvine, CA, United States; ^2^Department of Psychological Science, School of Social Ecology, University of California, Irvine, CA, United States; ^3^Department of Psychiatry, University of California San Diego, San Diego, CA, United States; ^4^Altman Clinical and Translational Research Institute, Dissemination and Implementation Science Center, University of California San Diego, San Diego, CA, United States; ^5^Child and Adolescent Services Research Center, San Diego, CA, United States; ^6^Sue & Bill Gross School of Nursing, University of California, Irvine, CA, United States

**Keywords:** loneliness, disability, online learning, mental health, higher education

## Abstract

**Background:**

People with disabilities face many health, economic and social disparities. Loneliness is recognized as a significant issue for this group however, its impact on students with disabilities (SWDs) remains a critically underexplored area of research. Importantly, as higher education continues its transition to the digital space, the potential to entrench social isolation and loneliness within this population has not been examined. This research seeks to explore the associations between SWDs, loneliness, online learning, and academic outcomes in a national survey of university students.

**Methods:**

Using the National College Health Assessment from Spring 2022, this study compared multiple outcomes between different groups of SWDs and students without disabilities. Two ordinal regression models were used to estimate associations between loneliness, disability type and learning mode (online vs. in-person), as well as cumulative grade average (CGA) with disability type.

**Results:**

All disability types included within this study were positively associated with increased odds of loneliness for those engaged in online learning compared to students without disabilities. Interactions indicate a significant effect of in-person learning compared with online learning for deaf or hard of hearing students, and those with multiple disabilities. Several disability groups reported decreased odds of higher CGA compared to students without disabilities.

**Conclusion:**

Loneliness is a significant issue for all SWD groups reported in this study. The impact of disability on academic outcomes is reported herein; however, its impact on medium-to-long term outcomes for these individuals remains unknown. To address inequities in higher education for SWDs, universities must invest more resources to holistically support these students.

## 1 Introduction

As much of the world has become increasingly digitalized, loneliness and social isolation have become significant concerns (Luhmann et al., 2023). This is true within higher education where the combination of transitioning to online learning in parallel with diminishing campus experiences has sparked concerns about increasing loneliness in young people (Lyons et al., 2020; Vakoufari et al., 2014). While digital tools facilitated continued learning within higher education during the pandemic, the impacts of this shift for different groups are not fully understood. Importantly, evidence during 2020 suggested that people aged 18–22 years may be the loneliest generation (The Cigna Group, 2020). Around a third-to-half of university students report loneliness during their university years (American College Health Association, 2023; Diehl et al., 2018) with estimates suggesting that this number is increasing (Hysing et al., 2020).

The relationship between loneliness and mental health in the general population and for those with disabilities is well understood (Fichten et al., 2014; Laslo-Roth et al., 2022; McIntyre et al., 2018). In student populations, loneliness is associated with higher anxiety, stress and depression (Diehl et al., 2018; Richardson et al., 2017) and poorer outcomes such as greater attrition and perception of success (Stoliker and Lafreniere, 2015). Previous studies have identified particular considerations for students with disabilities (SWDs) in relation to online learning, such as accessible infrastructure and staffing (Kent, 2015; Phillips et al., 2012), and also social and emotional supports (Kent et al., 2018; Zhang et al., 2022). In this context, loneliness has arisen as a significant concern for people with disabilities in the field of online learning.

Early evidence suggests that SWDs report higher levels of loneliness compared to those without disabilities (Laslo-Roth et al., 2022; Sharabi and Margalit, 2011). The physical remoteness of online learning disproportionately impacts SWDs where finding social support and making connections become more difficult in digital spaces (Kotera et al., 2021; McManus et al., 2017; Mizani et al., 2022; Zhang et al., 2022). Despite this, the impact of the increasing use of digital technologies in education on mental health outcomes in populations of SWDs remains unexplored. Greater understanding in this area is especially important for SWDs where the relationship between disability, mental health, and student outcomes are highly dependent on successful interactions between students and institutions (Chiu et al., 2019; Karmel and Nguyen, 2008). Therefore, online learning and digital infrastructure in higher education may be effective points-of-interventions to realize disability accessibility and equity (Fleming et al., 2017; Hoyle et al., 2022). To date, few studies have explored loneliness and health outcomes for people with disabilities, and these studies routinely lack comparator groups or are focused on one specific impairment group (Bailie et al., 2023). To better support SWDs succeed in higher education, a greater understanding of these associations are required.

This study aims to investigate the relationship between loneliness, online learning and learning outcomes in SWDs. Using National College Health Assessment survey data, we first investigate whether SWDs report loneliness at higher frequency compared to students without disability. Second, we examine the relationship between disability and its association with student grade outcomes across different groups of SWDs. This study tests the hypotheses that:

1. SWDs report increased odds of loneliness compared to students without disabilities dependent on levels of learning mode.
2. SWDs report increased odds of lower grade outcomes compared to students without disabilities.

## 2 Materials and methods

### 2.1 Data collection

This analysis used the American College Health Association-National College Health Assessment (ACHA-NCHA). The ACHA-NCHA originated in 2000 and is a national research survey designed to provide high-quality data on students’ health and wellbeing for use by policy makers and health educators. It is administered twice in an academic year: once in the Spring and once in the Fall semesters where interested institutions recruit their own students to participate in the Qualtrics survey. The data used here was the 2022 ACHA-NCHA III Spring edition, which iterated on the prior version II with the full elucidation of its development previously published (Lederer and Hoban, 2022). This sample consisted of 69,131 students from 129 public and private higher education institutions across the United States of America (American College Health Association, 2023).

### 2.2 Measures

#### 2.2.1 Outcomes

Loneliness was assessed using the UCLA Three-Item Loneliness Scale score (Hughes et al., 2004), a three-item scale that measure three dimensions of loneliness relational connectedness, social connectedness, and self-perceived isolation. Items are scored according to three responses: 1 (Hardly ever), 2 (Some of the time), 3 (Often). These items were summed for each respondent to yield a singular score, ranging from 3 to 9. Scores 3–5 have previously been considered “not lonely”, while 6 and above were considered “lonely”. (Steptoe et al., 2013); however this variable was treated as a continuous variable in this model per best practice (Altman and Royston, 2006). This scale has previously been used in different populations of young adults and students (Lee et al., 2023; Tulk et al., 2022), and is commonly used for those with disabilities (Bailie et al., 2023; McGlone and Long, 2020). Importantly, this scale assesses loneliness in the context of social isolation, and it has been validated and aligns well with the three items from the full in-person scale (Hughes et al., 2004).

Cumulative grade average (CGA) is the respondent’s grade average over the course of their studies to date. This is a self-report measure where students are presented with a list of grade letters from A+ to F and also ‘Not Applicable. The question was asked as ‘What is your approximate cumulative grade average’. This variable was treated as an ordinal variable within this analysis.

### 2.3 Primary exposure variables

Disability was measured by querying respondents ‘Do you have any of the following?’ where respondents could select multiple options from the following: Attention-Deficit/Hyperactivity Disorder (ADD or ADHD); autism spectrum disorder (ASD); deaf or hard of hearing (DHoH); learning disability; mobility/dexterity disability; blind/low Vision; speech or language disorder. Respondents were considered to have a disability if they checked one or more of these options. Learning mode (Entirely in-person; entirely online; mixed) was queried by asking ‘I am taking classes this term’ with three responses: Entirely in-person; entirely online; a mix of in-person and online classes.

### 2.4 Covariates

Covariates were selected based upon *a prior*i research investigating the relationship between loneliness, psychological distress and academic achievement in a university student population (Alyami et al., 2022; Bore et al., 2016; Mizani et al., 2022; Zhang et al., 2023). This included age, gender, race/ethnicity, visa status, and loneliness.

#### 2.4.1 Model 1

Age (continuous), gender (cismale; cisfemale; transmale; transfemale; nonbinary; genderqueer; agender; genderfluid; intersex; other/not listed), visa status (yes; no), race/ethnicity (White; Asian or Asian American; URM).

#### 2.4.2 Model 2

Age (continuous), loneliness (continuous), learning type (entirely online; entirely in-person), gender (cismale; cisfemale; transmale; transfemale; nonbinary; genderqueer; agender; genderfluid; intersex; other).

### 2.5 Data analysis

#### 2.5.1 Missing data

Data was assessed for missingness using base R functionalities to investigate missingness rates among included variables. Means (for continuous variables) and proportions (for categorical variables) were calculated and compared between missing and non-missing responses for disability, loneliness, CGA to determine whether missingness was completely at random or missing was not-at-random. Missingness was assumed as completely at random for this analysis and all variables described < 5% missingness rate.

Multivariate Imputation by Chained Equations (MICE) was used to impute missing data in this dataset utilizing the R package mice (Van Buuren and Groothuis-Oudshoorn, 2011). The methods used varies by variable class within R. For this analysis the methods used were: PMM (Predictive Mean Matching) for numeric variables, logistic regression for dichotomous variables, Bayesian polytomous regression for unordered categorical variables ≥ 2 levels, and proportional odds model for ordered categorical variables ≥ 2 levels. Due to the low number of missing data, 5–20 iterations may be appropriate to reach convergence and 20 was selected for these data (Van Buuren, 2018).

#### 2.5.2 Variable manipulation

Several levels of gender were combined into a single variable of ‘non-binary or other’ comprised of genderqueer, agender, genderfluid, intersex, non-binary and other/not identified. “Other” text responses were text-mined and any that were matched with existing categories were re-categorized to existing levels. The ‘mixed’ level of learning mode was excluded from analysis due to how it was queried whereby proportion of learning in-person and remote was not determined. Levels of race/ethnicity were collapsed into Underrepresented Minority (URM) group as defined across University of California campuses (Antonovics and Backes, 2013; Robinson et al., 2022; University of California, Riverside, 2024), which comprised of African American or Black, Hispanic/Latino/a/x, Middle Eastern/North African or Arab Origin, Native Hawaiian or Other Pacific Islander Native, American Indian or Native Alaskan. CGA variable levels were collapsed into letter names of A, B, C & D to reduce the number of outcome levels. Level F was excluded due to extremely small sample size (*n* = 52) and therefore, results were interpreted in the context of D as the floor. Selected “NA” level was also excluded within this analysis. Sensitivity analysis was performed between responses of “NA” and missing values within the sample to determine differences between the groups and determine further analyses to perform to explore these responses. This variable was reverse-coded for easier interpretation: A was the highest level for this variable (highest possible grade average) while D was the lowest level for this variable (lowest possible grade average).

#### 2.5.3 Statistical analysis

Participant characteristics were generated using descriptive statistics performed across all included variables (Table 1). The association between loneliness, and disability and learning type was assessed using an ordinal logistic regression model; the association between CGA and disability was assessed using an ordinal logistic regression model also. Both ordinal regression models used the MASS package within R (Venables and Ripley, 2002). Findings were reported as odds ratios (OR) for both model 1 and 2, reporting error as 95% CIs. Univariable and multivariable model results are both presented in tables however, only multivariable model coefficients are reported in-text. Proportional odds assumption (or the parallel regression assumption) was tested to determine whether the relationship between each pair of outcome groups is the same across these models. Statistical tests have previously been criticized for being prone to type 1 error (Harrell, 2001) and therefore, this analysis employs a graphical method to assess this assumption as described by the UCLA: Statistical Consulting Group (UCLA Statistical Consulting Group). The graph was produced using logit models to model the probability that the outcome (CGA) is greater than or equal to a value for each of its levels by comparing one predictor at a time. The assumption was assumed to hold if the distance between coefficients was similar across all estimates. Supplementary Figures 1, 2 describes the proportional odds output for these ordinal regression models. These data suggest the proportional odds assumption is met with slight deviations at extremes of some variables. Supplementary Figure 3 describes predicted probabilities of Model 1 to visualize levels of the interaction between disability type and learning type, which was generated using the ggeffects package in R (Lüdecke et al., 2024). Checks for multicollinearity were performed for bother ordinal regression models (Supplementary Tables 1, 2). Interactions between disability type and learning mode were hypothesized *a priori* and subsequently included in model 1.

**TABLE 1:** Participant characteristics stratified by disability type.

	Overall	None	ADD or ADHD	ASD	DHoH	Learning	Mobility	Blind/low vision	Speech	>1 Disability
	69131	52428	6479	559	697	765	279	1571	237	3682
Age (median (min-max))	21 (18–91)	21 (18–90)	21 (18–66)	21 (18–91)	22 (18–84)	21 (18–71)	21 (18–72)	20 (18–67)	20 (18–50)	21 (18–79)
**Gender (n, %)**										
Cis female	45294 (65.52)	35314 (67.36)	4087 (63.08)	197 (35.24)	419 (60.11)	583 (76.21)	185 (66.31)	1085 (69.06)	129 (54.43)	2011 (54.62)
Cis male	19567 (28.30)	15293 (29.17)	1725 (26.62)	187 (33.45)	241 (34.58)	138 (18.04)	58 (20.79)	419 (26.67)	92 (38.82)	840 (22.81)
Trans female	119 (0.17)	50 (0.10)	21 (0.32)	8 (1.43)	0 (0.00)	0 (0.00)	2 (0.72)	1 (0.06)	1 (0.42)	32 (0.87)
Trans male	253 (0.37)	108 (0.21)	42 (0.65)	14 (2.50)	1 (0.14)	1 (0.13)	1 (0.36)	4 (0.25)	3 (1.27)	69 (1.87)
Genderqueer	477 (0.69)	228 (0.43)	69 (1.06)	19 (3.40)	6 (0.86)	9 (1.18)	6 (2.15)	14 (0.89)	1 (0.42)	110 (2.99)
Identity not listed	358 (0.52)	181 (0.35)	55 (0.85)	12 (2.15)	4 (0.57)	6 (0.78)	0 (0.00)	8 (0.51)	3 (1.27)	70 (1.90)
Agender	215 (0.31)	90 (0.17)	31 (0.48)	24 (4.29)	1 (0.14)	2 (0.26)	4 (1.43)	4 (0.25)	0 (0.00)	55 (1.49)
Genderfluid	431 (0.62)	206 (0.39)	84 (1.30)	13 (2.33)	2 (0.29)	7 (0.92)	3 (1.08)	8 (0.51)	0 (0.00)	87 (2.36)
Intersex	1846 (2.67)	874 (1.67)	351 (5.42)	85 (15.21)	22 (3.16)	16 (2.09)	20 (7.17)	25 (1.59)	8 (3.38)	400 (10.86)
Non-binary	9 (0.01)	6 (0.01)	0 (0.00)	0 (0.00)	0 (0.00)	0 (0.00)	0 (0.00)	0 (0.00)	0 (0.00)	2 (0.05)
*Missing*	562 (0.81)	78 (0.15)	14 (0.22)	0 (0.00)	1 (0.14)	3 (0.39)	0 (0.00)	3 (0.19)	0 (0.00)	6 (0.16)
**Learning mode (n, %)**										
Entirely online	7013 (10.15)	5276 (10.06)	727 (11.22)	46 (8.23)	109 (15.64)	97 (12.68)	39 (13.98)	97 (6.17)	13 (5.49)	401 (10.89)
Entirely in-person	34516 (49.93)	26611 (50.76)	3073 (47.43)	306 (54.74)	302 (43.33)	383 (50.07)	132 (47.31)	816 (51.94)	127 (53.59)	1828 (49.65)
Mix	26902 (38.92)	20363 (38.84)	2651 (40.92)	202 (36.14)	286 (41.03)	284 (37.12)	107 (38.35)	654 (41.63)	96 (40.51)	1446 (39.27)
*Missing*	700 (1.01)	178 (0.34)	28 (0.43)	5 (0.89)	0 (0)	1 (0.13)	1 (0.36)	4 (0.25)	1 (0.42)	7 (0.19)
**Loneliness (n, %)**										
Not lonely	32967 (47.69)	26374 (50.31)	2779 (42.89)	165 (29.52)	345 (49.50)	337 (44.05)	107 (38.35)	662 (42.14)	97 (40.93)	1217 (33.05)
Lonely	35529 (51.39)	25890 (49.38)	3681 (56.81)	394 (70.48)	351 (50.36)	426 (55.69)	172 (61.65)	906 (57.67)	140 (59.07)	2457 (66.73)
*Missing*	635 (0.92)	164 (0.31)	19 (0.29)	0 (0.00)	1 (0.14)	2 (0.26)	0 (0.00)	3 (0.19)	0 (0.00)	8 (0.22)
**Race category (n, %)**										
White	42391 (61.30)	31656 (60.38)	4644 (71.68)	440 (78.71)	504 (72.31)	539 (70.46)	197 (70.61)	705 (44.88)	141 (59.49)	2531 (68.74)
American Indian or Native Alaskan	418 (0.61)	303 (0.58)	39 (0.60)	3 (0.54)	8 (1.15)	5 (0.65)	0 (0.00)	15 (0.95)	0 (0.00)	29 (0.79)
Asian or Asian American	8302 (12.0)	7118 (13.58)	301 (4.65)	17 (3.04)	52 (7.46)	31 (4.05)	11 (3.94)	292 (18.59)	22 (9.28)	189 (5.13)
Black or African American	3338 (4.83)	2709 (5.17)	187 (2.89)	13 (2.33)	16 (2.30)	37 (4.84)	9 (3.23)	107 (6.81)	20 (8.44)	124 (3.37)
Hispanic/Latino/a/x	5028 (7.27)	4030 (7.69)	338 (5.22)	17 (3.04)	36 (5.16)	48 (6.27)	17 (6.09)	189 (12.03)	12 (5.06)	183 (4.97)
Middle Eastern, North African or Arab Origin	728 (1.05)	573 (1.09)	56 (0.86)	1 (0.18)	3 (0.43)	4 (0.52)	5 (1.79)	25 (1.59)	2 (0.84)	28 (0.76)
Native Hawaiian or Other Pacific Islander	88 (0.13)	63 (0.12)	8 (0.12)	0 (0.00)	2 (0.29)	1 (0.13)	1 (0.36)	3 (0.19)	1 (0.42)	7 (0.19)
Two or more	7534 (10.90)	5386 (10.27)	839 (12.95)	63 (11.27)	65 (9.33)	89 (11.63)	35 (12.54)	217 (13.81)	33 (13.92)	545 (14.80)
Other	503 (0.73)	381 (0.73)	38 (0.59)	1 (0.18)	7 (1.00)	5 (0.65)	3 (1.08)	13 (0.83)	5 (2.11)	31 (0.84)
*Missing*	801 (1.16)	209 (0.40)	29 (0.45)	4 (0.72)	4 (0.57)	6 (0.78)	1 (0.36)	5 (0.32)	1 (0.42)	15 (0.41)
**Visa (n, %)**										
No	59979 (86.76)	45310 (86.42)	6055 (93.46)	521 (93.20)	632 (90.67)	693 (90.59)	263 (94.27)	1326 (84.40)	200 (84.39)	3402 (92.40)
Yes	8271 (11.96)	6875 (13.11)	391 (6.03)	34 (6.08)	61 (8.75)	68 (8.89)	16 (5.73)	236 (15.02)	36 (15.19)	261 (7.09)
*Missing*	881 (1.27)	243 (0.46)	33 (0.51)	4 (0.72)	4 (0.57)	4 (0.52)	0 (0.00)	9 (0.57)	1 (0.42)	19 (0.52)

## 3 Results

### 3.1 Participant characteristics

Table 1 provides sample characteristics across the entire cohort stratified by disability type. ADD or ADHD only was the most prevalent reported disability at 9.37% of the sample, while speech only was the least prevalent at 0.34% of the sample. A small percent (5.33%) of the sample reported having more than one disability; however, no combination of disabilities was more prevalent than any single disability. There were 2,434 missing responses for disability. Over half of all respondents reported feeling lonely (51.39%) while 70.48% those in the ASD group reported loneliness. The median age of overall sample was 21 years with a range of 18 to 91 years. Cis females accounted for the majority of respondents in each disability type except for those reporting ASD. Only 49.06% of respondents reported learning entirely or partially online compared to 49.93% who reported learning entirely in-person. Visa holders made up 11.96% of the overall cohort.

### 3.2 Regression models

#### 3.2.1 Model 1−loneliness

For those engaged in learning entirely online, all disabilities were associated with increased odds of higher loneliness compared to those reporting no disabilities: ADD or ADHD, ASD, DHoH, blind/low vision, learning, speech, mobility/dexterity and more than one disability (Table 2). For those without disabilities, learning entirely in-person was associated with a small increase in the odds of loneliness compared to learning entirely online. Interaction coefficients for loneliness between those who reported DHoH and more than one disability were significant, indicating a differential impact on the odds of loneliness for these groups engaged in entirely in-person learning compared with entirely online.

**TABLE 2:** Ordinal logistic regression of association between disability, learning mode, and loneliness (*N* = 40,643).

	DV = Loneliness	
**Variable**		
**Main effects**	**Main effects model only OR (95%CI)**	**Full adjusted OR (95% CI)**
*Disability:* None	Reference	Reference
ADD or ADHD only	1.44 (1.35–1.53)*	1.53 (1.33–1.76)*
Autism spectrum disorder only	2.78 (2.30–3.37)*	3.42 (2.00–5.84)*
Deaf or Hard of Hearing only	1.02 (0.85–1.22)	1.79 (1.21–2.63)*
Blind or low vision only	1.39 (1.18–1.63)*	1.52 (1.05–2.21)*
Learning only	1.78 (1.38–2.29)*	1.60 (1.10–2.34)*
Speech only	1.35 (1.20–1.52)*	3.65 (1.33–10.03)*
Mobility/dexterity only	1.58 (1.16–2.14)*	2.45 (1.44–4.16)*
More than one disability	2.21 (2.05–2.39)*	2.54 (2.11–3.05)*
*Learning mode:* Entirely online	Reference	Reference
Entirely in-person	1.37 (1.31–1.44)*	1.11 (1.05–1.18)*
**Interactions**	**Main effects model only OR (95%CI)**	**Adjusted ORR (95% CI)**
*Disability x Learning mode:* None x Entirely online	Reference	Reference
ADD or ADHD only x Entirely in-person	–	0.92 (0.79–1.07)
ASD only x Entirely in-person	–	0.66 (0.38–1.17)
DHoH only x Entirely in-person	–	0.60 (0.39–0.93)*
Blind or low vision only x Entirely in-person	–	0.79 (0.53–1.18)
Learning only x Entirely in-person	–	0.83 (0.55–1.26)
Speech only x Entirely in-person	–	0.38 (0.13–1.09)
Mobility/dexterity only x Entirely in-person	–	0.67 (0.37–1.23)
More than one disability x Entirely in-person	–	0.77 (0.63–0.94)*
**Confounders**	**Main effects model only OR (95%CI)**	**Full adjusted OR (95% CI)**
*Age:* Years	0.97 (0.97–0.97)*	0.97 (0.97–0.97)*
*Gender:* Female (cis)	Reference	Reference
Male (cis)	0.87 (0.84–0.91)*	0.87 (0.84–0.90)*
Female (trans)	2.21 (1.42–3.45)*	1.75 (1.11–2.74)*
Male (trans)	2.46 (1.87–3.24)*	1.83 (1.39–2.41)*
Non-binary or other	2.12 (1.96–2.29)*	1.70 (1.57–1.84)*
*Race category:* White	Reference	Reference
Asian or Asian American	1.17 (1.12–1.22)*	1.22 (1.15–1.29)*
Underrepresented minority	1.10 (1.04–1.16)*	1.22 (1.17–1.27)*
*Visa:* No	Reference	Reference
Yes	0.95 (0.90–1.00)	0.96 (0.91–1.02)

*p < 0.05.

#### 3.2.2 Model 2−CGA

Table 3 described those that reported ADD or ADHD, DHoH, blind/low vision, a learning disability or more than one disability were associated with decreased odds of being in a higher category of CGA compared to those without any disability for those learning entirely online when controlled for covariates.

**TABLE 3:** Ordinal logistic regression of association between disability and CGA (*N* = 40,643).

Variable	Main effects model only OR (95%CI)	Full adjusted OR (95% CI)
**Main effects**		
*Disability:* None	Reference	Reference
ADD or ADHD only	0.52 (0.49–0.56)*	0.52 (0.49–0.56)*
Autism spectrum disorder only	0.79 (0.63–0.98)*	0.89 (0.71–1.11)
Deaf or Hard of Hearing only	0.89 (0.73–1.09)	0.80 (0.65–0.99)*
Blind or low vision only	0.68 (0.59–0.78)*	0.71 (0.62–0.82)*
Learning only	0.56 (0.47–0.67)*	0.55 (0.46–0.66)*
Speech only	0.72 (0.51–1.01)	0.78 (0.55–1.10)
Mobility/dexterity only	1.09 (0.78–1.52)	1.05 (0.75–1.47)
More than one disability	0.44 (0.41–0.48)*	0.45 (0.41–0.49)*
**Confounders**		
*Learning mode:* Entirely online or mixed	Reference	Reference
Entirely in-person	0.73 (0.69–0.77)*	0.98 (0.92–1.05)
*Loneliness:* Yes	0.92 (0.91–0.93)*	0.94 (0.93–0.95)*
*Age*: Years	1.03 (1.03–1.04)*	1.03 (1.03–1.04)*
*Gender:* Female (cis)	Reference	Reference
Male (cis)	0.74 (0.71–0.78)*	0.72 (0.69–0.75)*
Female (trans)	0.59 (0.36–0.97)*	0.76 (0.46–1.26)
Male (trans)	0.68 (0.49–0.94)*	0.99 (0.71–1.38)
Non-binary or other	0.70 (0.64–0.77)*	0.93 (0.84–1.02)

*p < 0.05.

## 4 Discussion

The purpose of this study was to investigate the joint influence of disability and learning mode on loneliness, and the association between disability type and CGA. Using a nationally conducted survey of college students that assessed health behaviors and outcomes, this study reported increased loneliness for all disability groups for students studying entirely online. Specifically, the findings suggested that students undertaking entirely online learning across all reported disability categories reported increased odds of greater loneliness compared to those without disabilities. The significant interaction for students reporting DHoH and those with more than one disability suggested that these individuals had lower odds of experiencing loneliness when learning entirely in-person compared with learning entirely online. The results from model 2 described further differences across disability type. Several disability categories were associated with decreased odds of being in a higher CGA category compared to those without disabilities, and this effect was greatest in those with more than one disability.

The association of disability with loneliness is well-understood in many contexts (Bailie et al., 2023; Emerson et al., 2021; Emerson et al., 2023; Feldman et al., 2016; Macdonald et al., 2018; McVilly et al., 2006; Tarvainen, 2021), and the research interest in this relationship has grown over the last decade (Gómez-Zúñiga et al., 2023). In populations of students with and without disability, this relationship is less well-established; however, this study adds to the growing body of evidence of a direct association between experiences of disability and feelings of loneliness. Importantly, this is the first study to report significantly increased odds of loneliness across a range of disabilities in a large, national student database. Students who reported ASD or a speech disability had on average three times greater odds of greater loneliness compared to those without disabilities when learning entirely online. For those with a mobility/dexterity disability or those with more than one disability, it was more than double the odds. This difference between those reporting disabilities and those that do not, across such a large sample, suggests a significant divergence in the university experiences of these groups compared to students without disabilities. For students with autism spectrum disorder, our data are consistent with prior reports that also found loneliness as a significant burden (Ashbaugh et al., 2017; Hillier et al., 2018; Jackson et al., 2018). It is understood that university students with autism spectrum disorder experience significant social and mental health challenges such as depression, anxiety, and social isolation (Andersen, 1995; Gelbar et al., 2014; Jansen et al., 2018), which may exacerbate or be exacerbated by loneliness. In the context of these students reporting barriers to access and limitations associated with support services in academic environments (Davis et al., 2021; Pesonen et al., 2021), institutions must address these shortcomings to support this growing population of students (Elias and White, 2018).

There is disparate evidence around loneliness in college student populations for those who have a speech or mobility disability (Gelbar et al., 2015). Much of this may be a result of terminology where functional limitations in mobility or speech are considered symptoms of a broader disabling condition, such as multiple sclerosis or cerebral palsy, as opposed to the disability itself. Evidence does suggest that students with physical disabilities may feel disconnected from the broader university environment (Minotti et al., 2021), and individuals may feel a tension between being overly visible while also invisible (Abes and Wallace, 2018). One qualitative study described the experience of physical and social isolation associated with having a physical disability in a university environment (Kotera et al., 2021). However, little data is available for those with speech disabilities. Overall, our finding that loneliness existed across all disability groups indicates a critical need for better support within higher education institutions.

For those that reported DHoH and more than one disability, the significant interaction effect suggests that in-person learning was much less associated with increased loneliness compared to online learning. Importantly, visualizing this on the probability scale shows minor difference-in-differences for these groups, which suggests this effect is small (Supplementary Figure 3). In-person disability services and classroom accessibility for DHoH students has been improving over decades (Brett, 2010; Hyde et al., 2009; Millett, 2009), these data suggest that online learning environments may be lagging behind. Inversely, online learning for DHoH individuals has been associated with greater fatigue and worse performance outcomes (Rodrigues et al., 2022). In this context, the current study is novel in presenting the impact in terms of loneliness beyond the immediate COVID-19 period and describes an important consideration in the transition to online learning for those with DHoH and more than one disability in particular. Increasing the accessibility of online learning has been a long-term focus for DHoH students (Hagman, 2021; Mallory et al., 2003). Such efforts include understanding accessibility within videoconferencing software, online peer-to-peer instructional support and collaborative writing programs for blind/low vision individuals (Akter et al., 2023; Das et al., 2022; Saha et al., 2023). This and other studies provide evidence of continued challenges of accessibility to meet the needs of SWDs (Aljedaani et al., 2023). Importantly, more than one disability group contains many permutations of disability groups; it is possible that the effect of certain combinations of disabilities has a multiplicative effect on certain outcomes however, cross-tabulation is not feasible due to small sample sizes.

Other studies have suggested the disability and loneliness relationship is mediated by other variables such as mood (Sharabi and Margalit, 2011), mental distress (McIntyre et al., 2018), or perceived hope and social support (Laslo-Roth et al., 2022; Peltzer and Pengpid, 2017) in students. Mediation analysis was beyond the scope of this analysis; however, future studies should explore the complex relationship between disability and loneliness to explore possible points of intervention. This is especially important in the context of a potential dose-dependent response wherein this study having more than one disability was associated with an even greater odds of reporting loneliness compared with having only one disability.

In part, it is likely the corresponding isolation that may come with online learning that creates this additional vulnerability. Indeed, other previous studies have described the role of physical remoteness and technology in creating social isolation and alienation (Kotera et al., 2021; McManus et al., 2017). Students who learn online, particularly those with a disability, may not be able to find alternative outlets for social interaction, and as such may experience social isolation and alienation (Rokach, 2015). Prior studies have indicated that advantages of in-person or online learning for both students with and without disabilities (Zhang et al., 2022); however, to these authors’ knowledge, no study has explored feelings of loneliness. Furthermore, no studies to date have considered student preferences in a mixed learning approach, which is likely to be an important moderator. This is a critical knowledge gap considering the identified importance of flexible learning for SWDs where a mixed learning approach may support this need (Kotera et al., 2021; Zhang et al., 2022).

Overall, even despite increased odds of loneliness for all disability groups compared to those without, almost half of all students without disabilities in this sample reported loneliness within the last 12 months. These data are not used to calculate prevalence however, this suggests significantly increased loneliness in this student population within American universities compared to the general population (Surkalim et al., 2022). Universities are increasingly aware of this issue and continue to attempt to address this growing challenge however (Diehl et al., 2018; Ellard et al., 2023), results herein demonstrate that more attention is needed in this student population.

The academic outcomes result of this study indicates that the needs of SWDs are not being met where over half of disability groups reported decreased odds of higher CGA compared to those without disabilities. Previously reported differences in student outcomes vary across different disabilities and levels of education (DuPaul et al., 2021; Henning et al., 2022; Horn, 1999; Kilpatrick et al., 2017; Lombardi et al., 2016; Murray and Wren, 2003; Wessel et al., 2009), including the absence of a gap (Sachs and Schreuer, 2011; Stewart et al., 2013). This is a significant concern where it is well-established that barriers for success are determined by access to support services (Abreu et al., 2017; Chiu et al., 2019; Karmel and Nguyen, 2008; Rath and Royer, 2002), social supports (Carroll et al., 2020; Fleming et al., 2017; Lombardi et al., 2016) and transition to higher education from schooling (Foley, 2006; Lipka et al., 2020; Wray, 2013). To the authors’ knowledge, no study has described a link between academic outcomes and loneliness in student populations, and this relationship should be explored to further understand the impact of social isolation on academic outcomes. As previously mentioned, the association between distress and loneliness may play an important role, and this may be a point of intervention for disability services within universities. Ultimately, The Americans with Disabilities Act Title II and III regulations dictate that public and private colleges and universities are required to provide equal access to postsecondary education for SWDs (Ada.gov, 2012; Ada.gov, 2016) and therefore, disparities as described here must be addressed.

### 4.1 Limitations

This study has several limitations. Due to the cross-sectional nature of the dataset, temporal associations that may assist in explaining relationships between variables cannot be determined. It is also unclear how representative this dataset is to the general population of higher education students. Importantly, self-selection nature of participation may contribute to unrepresentativeness within these data where non-disclosure of disabilities is a common phenomenon. Analyzing disabilities as ‘only’ may not be representative of the way disability is experienced for many different individuals, particularly as this study report many combinations of disabilities. Low numbers for some groups prevent accurate estimations for cross-tabulation, as well as the limitation of model overfitting for > 7 disability types as a primary exposure with interactions. Gender identity groups faced similar issues where smaller groups were collapsed into ‘other’, which does not represent the underlying diversity reported by individuals. The exclusion of ‘mixed’ learning category may occlude important associations of students within this category. This exclusion was necessary due to the lack of specificity in which this question was asked whereby students learning 1% or 99% of their study load online may have been included in this group. Future studies should query this on a ‘percentage of study load undertaken online/in-person.’ These data are also self-reported, which has previously been identified as biasing for measures such as CGA wherein students are prone to over-report their grade average (Kuncel et al., 2005). As these analyses are comparative between certain groups, biasing may occur if certain groups are more likely to report than others; however, no such analysis has been undertaken. Similarly, disability may be under-or-overreported in this sample. Prior evidence suggests that SWDs underreport their disabilities to universities for many reasons (Eccles et al., 2018; Grimes et al., 2019); it is unclear if this is true within a self-report survey but it remains a possibility. Caution should be exercised in interpretation of coefficients of model 1 with wide confidence intervals, such as those in Speech and ASD groups, which indicates significant uncertainty around the estimate.

### 4.2 Conclusion

Loneliness remains a significant issue for higher education institutions with most of all respondents in this analysis, with and without disabilities, reporting loneliness. This study demonstrates an association of disabilities with increased loneliness compared to students without disabilities in a large, national, university student sample. Further, it demonstrates an association of SWDs with poorer student outcomes compared to students without disabilities in the context of online learning. This study highlights the need for higher education institutions to invest more resources into improving student wellbeing, particularly for SWDs who are more likely to experience loneliness compared to those without. This will not only lead to better student health outcomes but also to improved student academic outcomes.

## Data Availability

The data analyzed in this study is subject to the following licenses/restrictions: Membership of the American College Health Association. Requests to access these datasets should be directed to mhoban@acha.org.
