# Supplementary material for: Loneliness, online learning and student outcomes in college students living with disabilities: results from the National College Health Assessment Spring 2022
Source: Front Psychol. 2024 Oct 17;15:1408837. doi: 10.3389/fpsyg.2024.1408837 (PMC11525119; doi:10.3389/fpsyg.2024.1408837)
Supplement: Supplementary file 1 [file Data_Sheet_1.docx]

**Supplementary table 1. Multicollinearity check for Model 1**

|  | GVIF | Df | GVIF^(1/(2*Df)) |
| --- | --- | --- | --- |
| Disability type | 1.09 | 8 | 1.01 |
| Learning type | 1.38 | 1 | 1.18 |
| Race | 1.19 | 2 | 1.04 |
| Gender | 1.09 | 4 | 1.01 |
| Age | 1.38 | 1 | 1.17 |
| Visa | 1.19 | 1 | 1.09 |

**Supplementary table 2. Multicollinearity check for Model 2**

|  | GVIF | Df | GVIF^(1/(2*Df)) |
| --- | --- | --- | --- |
| Disability type | 1.09 | 8 | 1.01 |
| Learning type | 1.33 | 1 | 1.15 |
| Loneliness | 1.03 | 1 | 1.02 |
| Gender | 1.09 | 4 | 1.01 |
| Age | 1.34 | 1 | 1.16 |
