# Supplementary material for: Loneliness, online learning and student outcomes in college students living with disabilities: results from the National College Health Assessment Spring 2022
Source: Front Psychol. 2024 Oct 17;15:1408837. doi: 10.3389/fpsyg.2024.1408837 (PMC11525119; doi:10.3389/fpsyg.2024.1408837)

Supplemental Figure 1. Test of proportional odds for ordinal regression model 1: loneliness

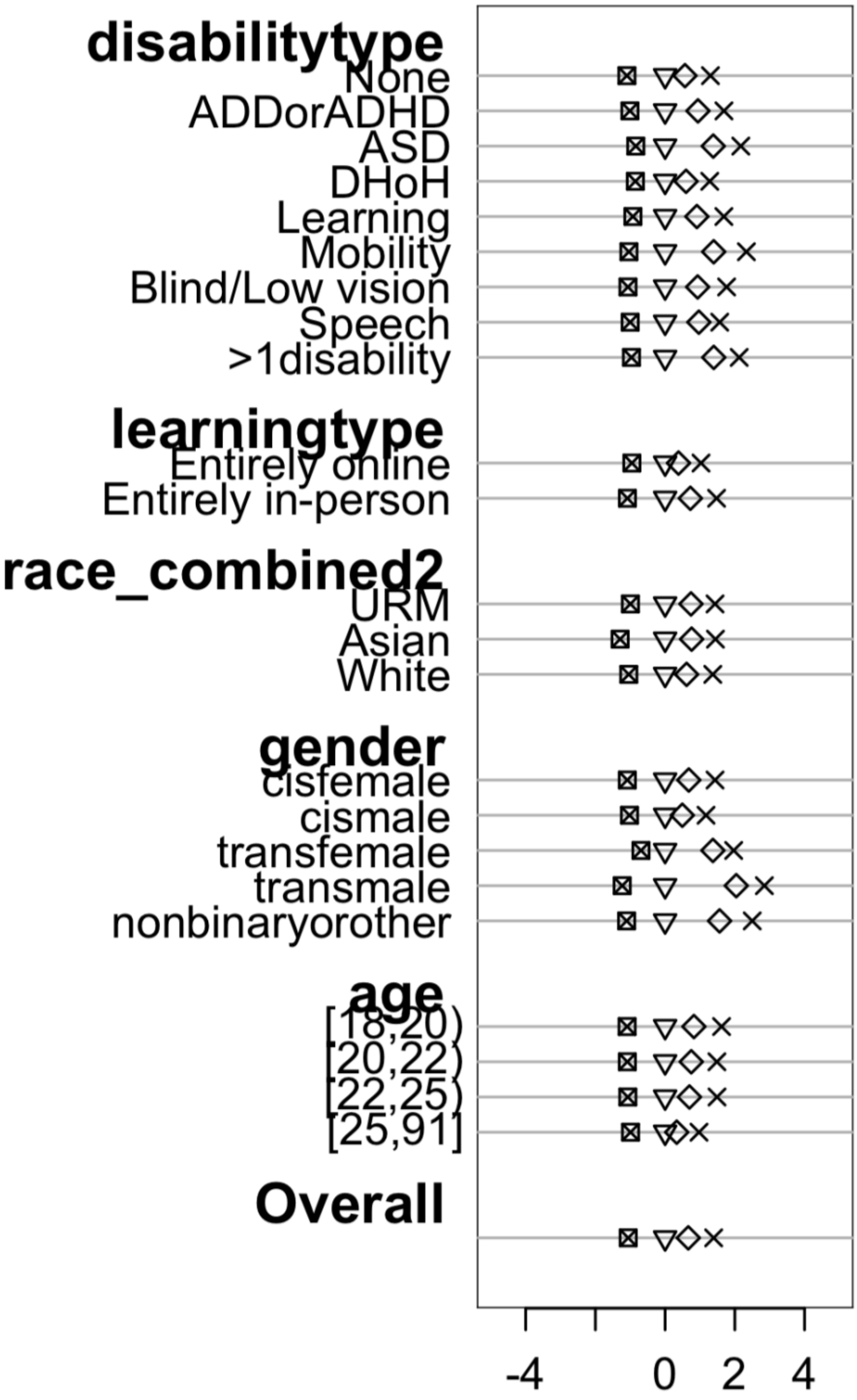

Supplemental Figure 2. Test of proportional odds for ordinal regression model 2: CGA.

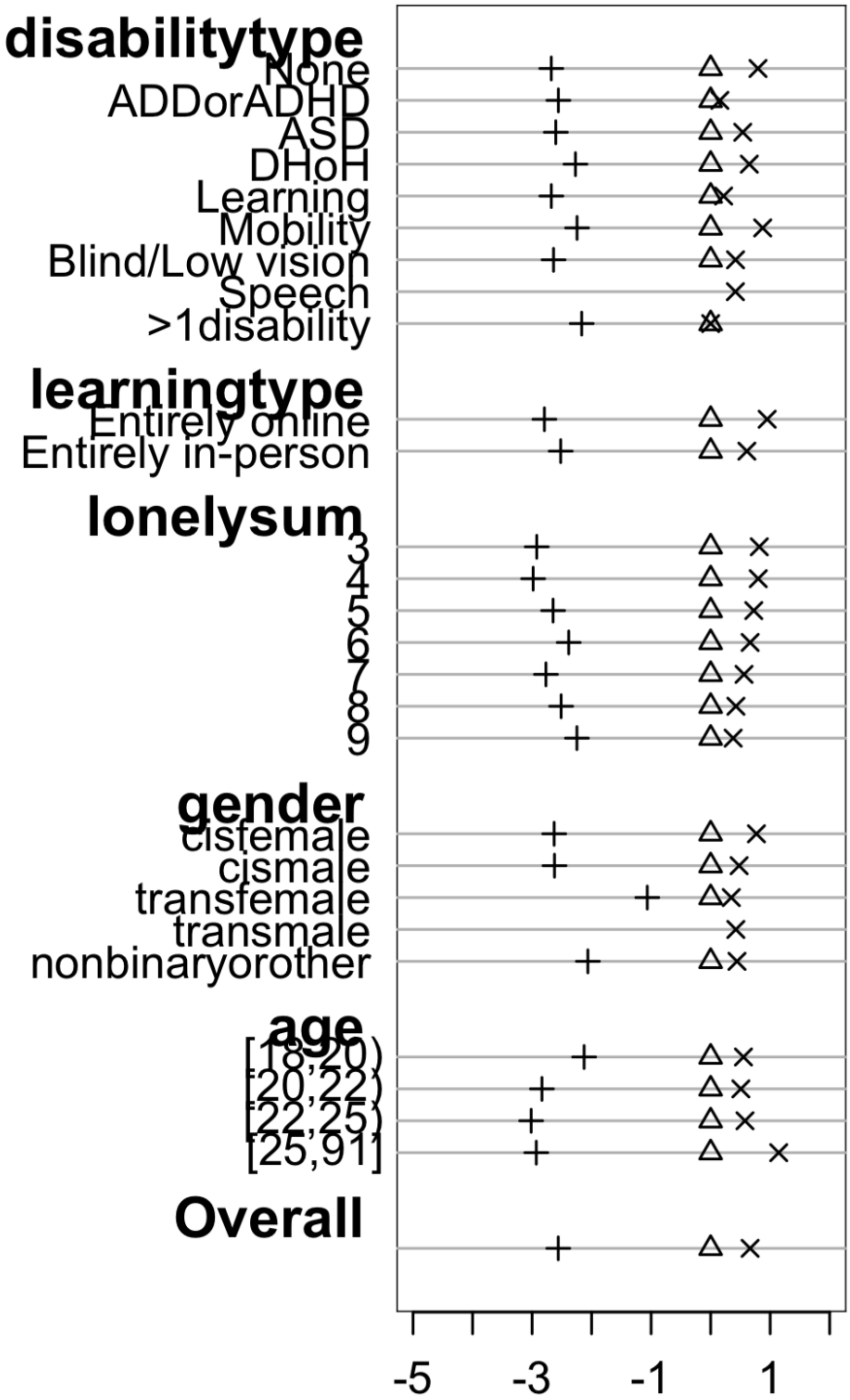

Supplemental Figure 3. Predicted probability plots of loneliness for all disability types by learning type at covariate means.

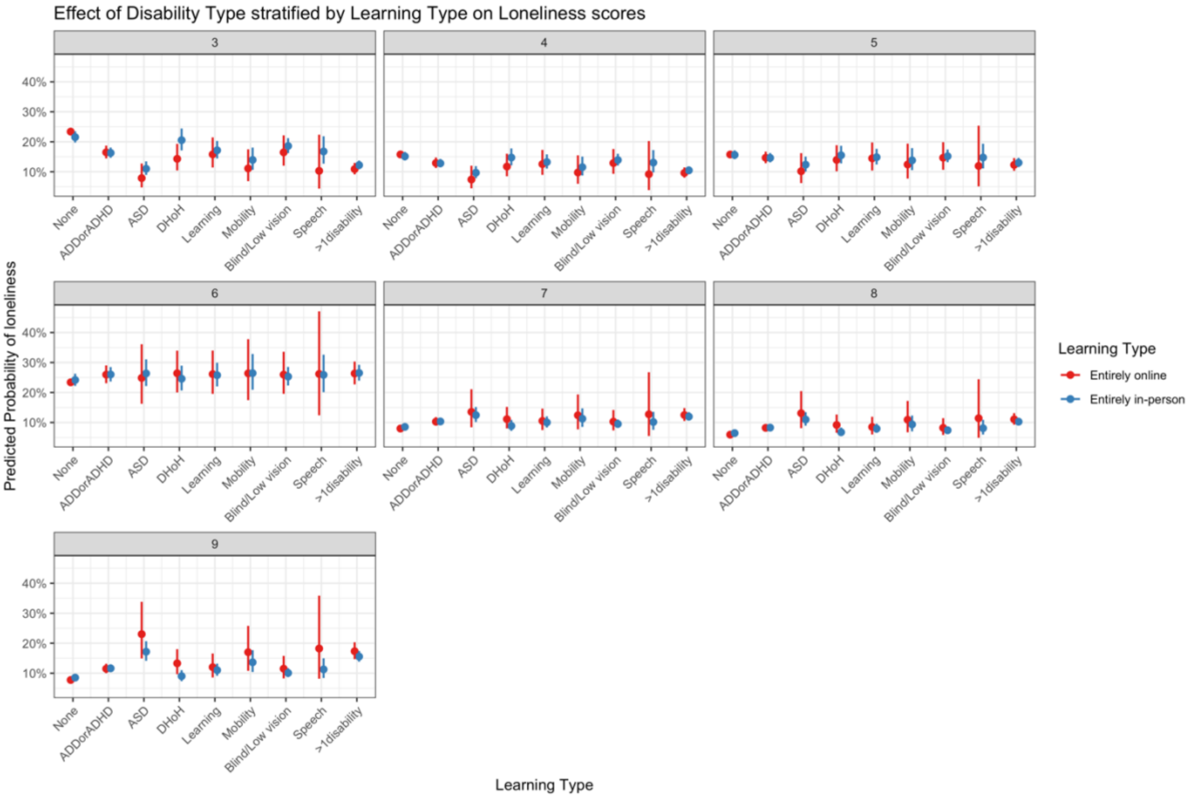

Supplement: Supplementary file 2 [file Image_1.pdf]
